# Clinical characteristics of patients with asymptomatic and symptomatic COVID-19 admitted to a tertiary referral centre in the Philippines

**DOI:** 10.1016/j.ijregi.2022.02.002

**Published:** 2022-02-06

**Authors:** Anna Flor G. Malundo, Cybele Lara R. Abad, Maria Sonia S. Salamat, Joanne Carmela M. Sandejas, Jose Eladio G. Planta, Jonnel B. Poblete, Shayne Julieane L. Morales, Ron Rafael W. Gabunada, Agnes Lorrainne M. Evasan, Johanna Patricia A. Cañal, Julian A. Santos, Jeffrey T. Manto, Raniv D. Rojo, Eric David B. Ornos, Mary Elise L. Severino, Maria Elizabeth P. Mercado, Marissa M. Alejandria

**Affiliations:** a Division of Infectious Diseases, University of the Philippines–Philippine General Hospital, Manila, Philippines; b Department of Medicine, University of the Philippines–Philippine General Hospital, Manila, Philippines; c Department of Radiology, University of the Philippines–Philippine General Hospital, Manila, Philippines; d College of Medicine, University of the Philippines, Manila, Philippines; e Department of Clinical Epidemiology, Faculty of Medicine and Surgery, University of Santo Tomas, Manila, Philippines

**Keywords:** COVID-19, Philippines, Epidemiology, Asymptomatic, Severity

## Abstract

**Objectives:**

To describe the clinical profile and outcomes of hospitalized patients with coronavirus disease 2019 (COVID-19) across the spectrum of disease severity.

**Methods:**

This retrospective study included adult patients with confirmed COVID-19 infection admitted to a referral hospital. Descriptive statistics, tests for trend, Kaplan–Meier curve and log-rank test were used to compare characteristics and outcomes across disease severity categories.

**Results:**

Of 1500 patients with COVID-19, 14.8% were asymptomatic, 13.5% had mild disease, 36.6% had moderate disease, 12.3% had severe disease and 22.7% had critical disease. Asymptomatic patients were admitted for a concurrent condition or for isolation. Patients aged >60 years, male gender and with co-morbidities had more severe disease. Fever, cough, shortness of breath, malaise, gastrointestinal symptoms and decreased sensorium were more common in patients with severe disease. Bilateral pulmonary infiltrates were common (51.1%), with sicker patients having more abnormal findings. The overall mortality rate was 15.1%. Adopting a symptom-based strategy reduced the length of hospitalization from a median of 13 [interquartile range (IQR) 7–21] days to 9 (IQR 5–14) days.

**Conclusion:**

The clinical profile and outcomes for this cohort of patients with COVID-19 was consistent with published reports. Asymptomatic infection was common, and universal testing may be a valuable strategy in the correct context, given the implications for infection control. A symptom-based strategy was found to reduce the length of hospitalization considerably.

## Highlights

• Asymptomatic infection is common.
• Bimodal age distribution of coronavirus disease 2019 (COVID-19) was observed at the University of the Philippines–Philippine General Hospital.
• Universal testing impacts infection control measures in resource-limited settings.
• Further blood testing is likely to be unnecessary for mild and asymptomatic cases of COVID-19.
• Symptom-based isolation protocol reduces length of hospitalization.

## Introduction

Coronavirus disease 2019 (COVID-19) is caused by severe acute respiratory syndrome coronavirus 2 (SARS-CoV-2), and has resulted in a pandemic infecting more than 273 million people worldwide (World Health Organization, 2021a). With the pandemic in its second year, much research has been undertaken to understand viral pathogenesis, transmission, and risk factors for disease progression and mortality (World Health Organization, 2021b). Governments and hospitals have adapted to structural and resource limitations with changes in policies. However, despite this, the Philippines remains the most affected country in the Western Pacific Region, reporting the highest cumulative number of cases of COVID-19 and associated deaths as of 22 December 2021 (World Health Organization, 2021c).

Most published data on COVID-19 have originated from Western and high-income countries. The largest published study from the Philippines is a multi-centre study with 10,881 participants, which focused on neurologic manifestations among patients with COVID-19 (Espiritu et al., 2021). Another study investigated 500 patients from an infectious disease referral hospital in Manila, and compared the clinical profile and outcomes among healthcare workers (HCWs) and non-HCWs (Agrupis et al., 2021). Other studies from the Philippines described experiences early in the pandemic, had small study sizes, and excluded asymptomatic individuals (Edrada et al., 2020; Abad et al., 2021; Salamat et al., 2021; Soria et al., 2021). Approximately 25% of cases of COVID-19 are asymptomatic (Alene et al., 2021), and this population has the potential to drive community transmission and strain healthcare resources. At the University of the Philippines–Philippine General Hospital (UP-PGH), asymptomatic infections are often identified through universal testing (i.e. SARS-CoV-2 testing regardless of indication for admission or presence of symptoms). Due to uncertainties early in the pandemic, patients with mild COVID-19 and asymptomatic patients were sometimes admitted for monitoring. Aside from the excessive admissions, hospital capacity was also strained by implementation of the test-based strategy, which required two consecutive negative SARS-CoV-2 test results before discontinuation of transmission-based precautions and discharge of recovered patients. This policy was updated in July 2021, such that specific groups of patients could be discharged as long as significant clinical recovery was noted, and antipyretics were not required during the last 3 days of a 14-day isolation period (i.e. symptom-based strategy) (Philippine Society for Microbiology and Infectious Diseases et al., 2020).

This study aimed to describe the clinical characteristics and outcomes of the first 1500 adult inpatients with confirmed COVID-19 at UP-PGH with a view to prioritizing healthcare resources. The clinical predictors of mortality and disease progression have been analyzed in a separate paper.

## Methods

### Study design and setting

The medical records of adult inpatients with confirmed COVID-19 infection at UP-PGH, a tertiary-level COVID-19 referral hospital in Manila, the epicentre of the COVID-19 pandemic in the Philippines, were reviewed. The UP-Manila Ethics Review Board approved this study, including waiver of patient informed consent.

### Study sample

**Figure 1:**
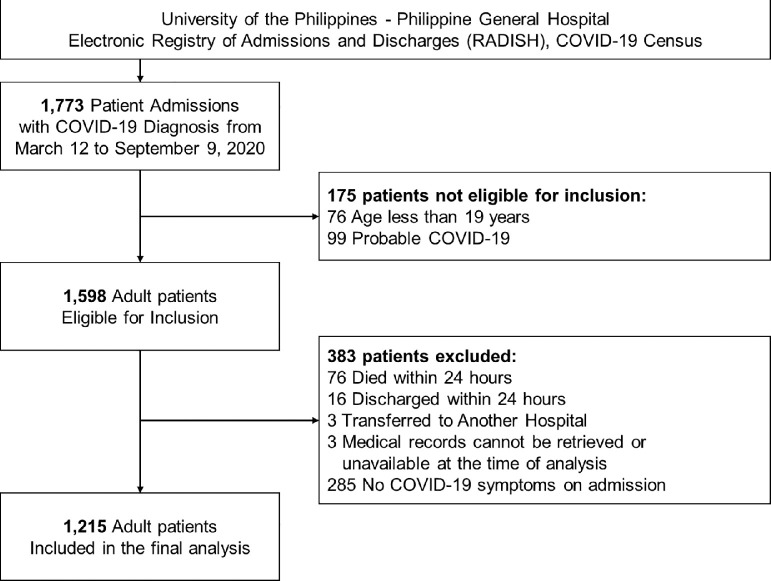
Flow chart of patient selection process. COVID-19, coronavirus disease 2019.

The UP-PGH Registry of Admissions and Discharges was used to identify patients aged ≥19 years with confirmed COVID-19. Due to limited clinical information, patients who died or were discharged within 24 h of admission, patients who were transferred to another hospital, and patients whose medical records were unavailable for review during the time of analysis were excluded from this study. In total, 1500 consecutive inpatients from 12 March 2020 to 9 September 2020 with confirmed COVID-19 were included in this study Figure 1. shows the cohort selection process.

### Definitions

A confirmed case of COVID-19 was defined as any patient with a positive reverse transcription polymerase chain reaction (RT-PCR) test for SARS-CoV-2 conducted at a testing facility accredited by the Department of Health. RT-PCR, which detects viral RNA in respiratory samples, is the current standard for the confirmation of acute SARS-CoV-2 infection (World Health Organization, 2020). RT-PCR has reported sensitivity of 0.68 (probability interval 0.63–0.73) and specificity of 0.99 (probability interval 0.98–1.00) (Kostoulas et al., 2021). False-negative results may be caused by poor-quality specimens; specimens taken late in the course of disease; inappropriate handling of specimens; and technical reasons, such as PCR inhibition or viral mutation (World Health Organization, 2020).

Severity of disease was classified at hospital admission, as follows:

• Asymptomatic – absence of COVID-19 symptoms and no evidence of pneumonia.
• Mild – presence of COVID-19 symptoms but no evidence of pneumonia.
• Moderate – presence of COVID-19 symptoms, and co-morbidities such as hypertension, cardiovascular disease, diabetes mellitus, chronic obstructive pulmonary disease (COPD), asthma, immunocompromising conditions (e.g. human immunodeficiency virus infection, chronic steroid use, active malignancy); or pneumonia but without need for oxygen support.
• Severe – presence of pneumonia, oxygen saturation ≤92% on room air and requiring oxygen support.
• Critical – acute respiratory distress syndrome, septic shock, on mechanical ventilation or admitted to the intensive care unit (Philippine Society for Microbiology and Infectious Diseases et al., 2020).

In-hospital mortality was defined as death during hospitalization, regardless of cause. Mortality rate was calculated as the number of deaths divided by the total number of cases.

### Data collection

A group of physicians extracted data from medical records using a standard electronic data collection form. Chest x-ray images were reviewed by three radiologists on the study team. Missing data, inconsistencies and data accuracy were reviewed. Conflicting data were resolved by consensus.

Demographic data and information regarding co-morbid diseases, reasons for admission of asymptomatic patients, symptoms on presentation, vital signs on admission, severity of disease on admission, and results of diagnostic studies were collected. Outcomes such as length of hospital stay and mortality were recorded.

### Data analysis

Counts and percentages were used to summarize categorical variables. The Shapiro–Wilk test was used to assess normality of continuous variables, and data are expressed as median and interquartile range (IQR), as appropriate. Univariate analyses, with the Cochran–Armitage test for trend for categorical variables and the Jonckheere–Terpstra trend test for continuous variables, were performed to compare parameters across the spectrum of disease severity. Post-hoc test (Conover) was performed for pairwise comparison of groups. The length of hospital stay was compared by severity of disease, and after revision of the discharge criteria. Kaplan–Meier analysis with log-rank test was performed to compare 28-day mortality across the spectrum of disease severity categories.

All tests were two-tailed, with *P*<0.05 considered to indicate statistical significance. Analyses were conducted using Microsoft Excel and MedCalc Statistical Software Version 19.7.4.

## Results

### Clinical characteristics

**Figure 2:**
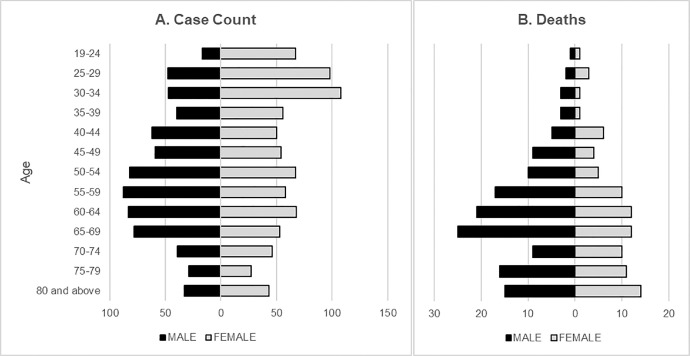
Age and sex distribution of 1500 patients with confirmed coronavirus disease 2019 admitted to the University of the Philippines–Philippine General Hospital. Black bars, males; grey bars, females.

Of the 1500 participants, 53.0% were female (*n*=795) and 66.7% were aged <60 years (*n*=1001). The median age was 51 (IQR 34–63) years, with a bimodal age distribution as shown in Figure 2. More than half of the participants had co-morbid conditions (*n*=961, 64.1%), with hypertension being the most common (*n*=628, 41.9%). Three hundred and five (20.3%) participants were HCWs, and 12.3% (*n*=185) were pregnant. The demographic and clinical profile of the study cohort is shown in Table 1.

**Table 1:** Demographic and clinical profile of inpatients with confirmed coronavirus disease 2019 stratified based on severity of disease.

	Asymptomatic (*n*=222)	Mild (*n*=203)	Moderate (*n*=549)	Severe (*n*=185)	Critical (*n*=341)	*P*-value
Age, years						
Median (IQR)	33 (27–38)	31 (28–42)	54 (43–64)	57 (46–67)	63 (53–71)	<0.01
≥60, *n* (%)	14 (6.3)	0 (0)	206 (37.5)	81 (43.8)	198 (58.1)	<0.01
Sex, *n* (%)						
Male	45 (20.3)	79 (38.9)	267 (48.6)	104 (56.2)	210 (61.6)	<0.01
Co-existing condition, *n* (%)						
Any co-morbid illness	55 (24.8)	54 (26.6)	433 (78.9)	141 (76.2)	278 (81.5)	<0.01
Diabetes mellitus	10 (4.5)	0 (0)	158 (28.8)	59 (31.9)	102 (29.9)	<0.01
Hypertension	29 (13.1)	0 (0)	304 (55.4)	96 (51.9)	199 (58.4)	<0.01
Heart disease	1 (0.5)	0 (0)	84 (15.3)	30 (16.2)	55 (16.1)	<0.01
Chronic liver disease	1 (0.5)	1 (0.5)	5 (0.9)	0 (0)	5 (1.5)	0.25
Chronic kidney disease	2 (0.9)	2 (1.0)	57 (10.4)	29 (15.7)	24 (7.0)	<0.01
COPD	0 (0)	0 (0)	10 (1.8)	6 (3.2)	13 (3.8)	<0.01
Asthma	3 (1.4)	24 (11.8)	36 (6.6)	12 (6.5)	16 (4.7)	0.96
Active PTB	0 (0)	1 (0.5)	15 (2.7)	10 (5.4)	14 (4.1)	<0.01
HIV	1 (0.5)	0 (0)	7 (1.3)	0 (0)	0 (0)	0.39
Cancer	12 (5.4)	0 (0)	39 (7.1)	9 (4.9)	28 (8.2)	0.02
Neurologic disease	2 (0.9)	1 (0.5)	30 (5.5)	17 (9.2)	39 (11.4)	<0.01
Smoker	14 (6.3)	19 (9.4)	107 (19.5)	39 (21.1)	98 (28.7)	<0.01
Drinker (alcohol)	19 (8.6)	40 (19.7)	111 (20.2)	46 (24.9)	102 (29.9)	<0.01
History of illicit drug use	1 (0.5)	3 (1.5)	9 (1.6)	5 (2.7)	6 (1.8)	0.21
Special population, *n* (%)						
Healthcare workers	44 (19.8)	125 (61.6)	111 (20.2)	14 (7.6)	11 (3.2)	<0.01
Pregnant women	132 (59.5)	8 (3.9)	39 (7.1)	4 (2.2)	2 (0.6)	<0.01

COPD, chronic obstructive pulmonary disease; PTB, pulmonary tuberculosis; IQR, interquartile range.

Of the study cohort, 81.0% (*n*=1215) were symptomatic and 19.0% (*n*=285) did not present with any symptoms suggestive of COVID-19. Of the latter, 215 (75.4%) were found to be COVID-19 positive on routine screening. Reasons for admission included obstetric management (*n*=162), surgery or procedure (*n*=17), cancer evaluation or chemotherapy (*n*=15), trauma (*n*=11) and other medical conditions (*n*=10). The remaining 70 patients were tested for SARS-CoV-2 as part of contact tracing (*n*=28), hospital surveillance (*n*=24), travel requirement (*n*=8), pre-employment (*n*=6) or for haemodialysis (*n*=4). However, 63 of the 285 (22.1%) patients who did not have COVID-19 symptoms were found to have subclinical disease with pneumonia on chest x-ray (*n*=61) or hypoxaemia requiring oxygen support (*n*=2). As a result, only 14.8% (*n*=222) of participants were assessed to be asymptomatic, while 13.5% (*n*=203), 36.6% (*n*=549), 12.3% (*n*=185) and 22.7% (*n*=341) had mild, moderate, severe and critical COVID-19, respectively.

Severity of disease increased progressively with age (Table 1). Males, smokers, drinkers (alcohol) and patients with co-morbidities (e.g. diabetes mellitus, hypertension, heart disease, chronic kidney disease, COPD, active pulmonary tuberculosis, cancer, pre-existing neurologic disease) tended to have more severe disease Table 2. shows that the majority of HCWs and pregnant women were asymptomatic or had mild disease. Furthermore, most of these patients were aged <60 years and did not have any co-morbidities.

**Table 2:** Clinical profile and outcomes of healthcare workers and pregnant inpatients with coronavirus disease 2019 at the University of the Philippines–Philippine General Hospital.

	Healthcare workers (*n*=305)	Pregnant (*n*=185)
Age, years		
Median (IQR)	39 (29–51)	30 (25–34)
<60, *n* (%)	283 (92.8)	185 (100)
≥60, *n* (%)	22 (7.2)	0 (0)
Sex, *n* (%)		
Male	126 (41.3)	Not applicable
Concurrent conditions, *n* (%)		
Presence of any co-morbid illness	138 (45.2)	30 (16.2)
Smoker	25 (8.2)	6 (3.2)
Drinker (alcohol)	50 (16.4)	7 (3.8)
History of illicit drug use	1 (0.3)	1 (0.5)
Disease activity, *n* (%)		
Asymptomatic	44 (14.4)	132 (71.4)
Mild	125 (41.0)	8 (4.3)
Moderate	111 (36.4)	39 (21.1)
Severe	14 (4.6)	4 (2.2)
Critical	11 (3.6)	2 (1.1)
Mortality, *n* (%)	3 (1.0)	1 (0.5)
Length of hospital stay, days, median (IQR)	11 (8–14)	3 (2–5)

IQR, interquartile range.

Commonly reported symptoms were cough (50.1%), fever (46.9%) and shortness of breath (37.2%). The frequency of fever, cough, shortness of breath, malaise, nausea or vomiting, decreased appetite, diarrhoea, abdominal pain or discomfort, and decreased sensorium increased with severity of disease (Table 3; see Table S1 in the online supplementary material for comprehensive list of symptoms). Abnormal vital signs on admission were observed among those with severe and critical COVID-19, with higher respiratory rates, lower peripheral oxygen saturation and lower Glasgow Coma Scale scores (Table 3).

**Table 3:** Clinical characteristics on admission and outcomes of patients with coronavirus disease 2019 at University of the Philippines–Philippine General Hospital.

	Severity of disease on admission					*P*-value
	Asymptomatic (*n*=222)	Mild (*n*=203)	Moderate (*n*=549)	Severe (*n*=185)	Critical (*n*=341)	
Symptoms, *n* (%)						
Fever	-	97 (47.8)	269 (49.0)	108 (58.4)	229 (67.2)	<0.001
Cough	-	91 (44.8)	285 (51.9)	132 (71.4)	244 (71.6)	<0.001
Shortness of breath	-	40 (19.7)	145 (26.4)	130 (70.3)	243 (71.3)	<0.001
Malaise/fatigue/						
generalized weakness	-	50 (24.6)	132 (24.0)	58 (31.4)	105 (30.8)	<0.001
Diarrhoea	-	44 (21.7)	76 (13.8)	27 (14.6)	52 (15.2)	<0.001
Nausea or vomiting	-	7 (3.4)	23 (4.2)	14 (7.6)	21 (6.2)	<0.001
Decreased appetite	-	9 (4.4)	44 (8.0)	33 (17.8)	75 (22.0)	<0.001
Abdominal pain/discomfort	-	9 (4.4)	26 (4.7)	9 (4.9)	14 (4.1)	0.048
Change or loss in taste	-	17 (8.4)	39 (7.1)	14 (7.6)	24 (7.0)	0.012
Decreased sensorium^c^	-	5 (2.5)	23 (4.2)	7 (3.8)	53 (15.5)	<0.001
Vital signs, median (IQR)						
Systolic blood pressure, mmHg	120 (110–130)	120 (110–130)	130 (120–140)	130 (120–144)	130 (113–145)	<0.001
Diastolic blood pressure, mmHg	80 (70–80)	78 (70–80)	80 (70–86)	80 (70–85)	80 (70–85)	0.069
Heart rate, beats/min	85 (78–92)	83 (76–91)	84 (78–92)	90 (80–102)	98 85–112)	<0.001
Respiratory rate, breaths/min	20 (19–20)	20 (18–20)	20 (20–20)	22 (20–24)	26 (23–30)	<0.001
Temperature, ^o^C	36.6 (36.4–36.9)	36.5 (36.1–36.8)	36.6 (36.2–37.0)	36.7 (36.5–37.3)	36.7 (36.3–37.0)	<0.001
Peripheral O_2_ saturation, %	98 (97–99)	98 (97–99)	98 (96–98)	95 (91–97)	92 (81–96)	<0.001
Glasgow Coma Scale score	15 (15–15)	15 (15–15)	15 (15–15)	15 (15–15)	15 (15–15)	<0.001
Laboratory findings						
*Complete blood count*, median (IQR)						
Haemoglobin, g/L	127 (117–137)	140 (132–149)	130 (114–143)	124 (105–138)	128 (110–143)	<0.001
White blood cells, x10^9^/L	10.3 (8.0–12.7)	7.1 (5.2–8.9)	7.2 (5.5–9.4)	7.8 (5.8–10.3)	10.6 (7.4–14.9)	<0.001
Neutrophils, %	70.0 (63.3–77.0)	59.0 (51.0–66.0)	65.0 (56.0–74.0)	76.0 (69.0–83.0)	84.0 (76.0–89.0)	<0.001
ALC, x10^9^/L	1.97 (1.57–2.48)	1.96 (1.56–2.44)	1.53 (1.09–2.06)	1.00 (0.80–1.35)	0.91 (0.58–1.39)	<0.001
Platelets, x10^9^/L	284 (237–347)	293 (238–336)	271 (202–364)	270 (181–385)	267 (203–343)	0.009
*Arterial blood gas*, median (IQR)						
pO_2_ and FiO_2_ ratio	467 (424–511)	467 (429–519)	410 (360–462)	338 (281–425)	175 (108–262)	<0.001
*Blood chemistry*, median (IQR)						
Serum creatinine, µmol/L	56.0 (46.0–74.5)	61.5 (52.0–76.5)	70.0 (54.0–97.0)	80.0 (60.0–127.3)	93.0 (69.0–165.5)	<0.001
Alanine aminotransferase, IU/L	18.0 (12.0–34.0)	30.0 (17.0–54.5)	31.5 (21.0–67.0)	45.0 (21.0–80.0)	46.0 (26.0–79.3)	<0.001
Albumin, g/L	39.0 (35.8–44.0)	45.0 (41.0–47.0)	38.0 (34.0–43.0)	35.0 (32.0–38.0)	34.0 (30.0–38.0)	<0.001
Total bilirubin, mg/dL	0.57 (0.42–0.87)	0.55 (0.44–0.77)	0.63 (0.49–0.89)	0.76 (0.49–0.99)	0.86 (0.58–1.20)	<0.001
*Inflammatory markers*, median (IQR)						
Lactate dehydrogenase, U/L	233 (203–279)	220 (187–253)	281 (230–353)	374 (307–487)	541 (390–748)	<0.001
Serum ferritin, ng/mL	89 (38–229)	126 (56–330)	407 (196–806)	1000 (460–1950)	1280 (704–2280)	<0.001
Serum procalcitonin, ng/mL	0.07 (0.04–0.11)	0.04 (0.04–0.05)	0.07 (0.04–0.28)	0.24 (0.09–1.04)	0.47 (0.17–1.65)	<0.001
D-dimer, µg/mL	0.62 (0.32–1.42)	0.40 (0.30–0.74)	0.82 (0.42–1.86)	1.68 (0.87–3.23)	2.74 (1.44–7.52)	<0.001
C-reactive protein, *n* (%)						
Not tested	129 (58.1)	31 (15.3)	109 (19.9)	23 (12.4)	30 (8.8)	
≤12 mg/L	84 (37.8)	143 (70.4)	221 (40.3)	25 (13.5)	24 (7.0)	
>12 mg/L	9 (4.1)	29 (14.3)	219 (39.9)	137 (74.1)	287 (84.2)	<0.001
*Chest radiograph, n (%)*						
No chest x-ray^a^	69 (31.1)	3 (1.5)	2 (0.4)	1 (0.5)	1 (0.3)	
*Pulmonary infiltrates*						
Bilateral	0 (0)	0 (0)	261 (47.5)	160 (86.5)	306 (89.7)	<0.001
>50% of lungs	0 (0)	0 (0)	109 (19.9)	129 (69.7)	261 (76.5)	<0.001
Limited–periphery	0 (0)	0 (0)	52 (9.5)	16 (8.6)	17 (5.0)	0.011
*Density*						
Ground glass^b^	0 (0)	0 (0)	244 (44.4)	134 (72.4)	269 (78.9)	<0.001
Consolidation	0 (0)	0 (0)	13 (2.4)	22 (11.9)	68 (19.9)	<0.001
Mortality, *n* (%)	1 (0.5)	0 (0)	34 (6.2)	29 (15.7)	162 (47.5)	<0.001
Length of hospital stay, days,						
median (IQR)	4 (3–9)	11 (7–14)	12 (8–20)	15 (10–24)	14 (7–21)	<0.001
Before change in guidelines^d^	7 (4–12)	12 (7–17)	13 (9–23)	16 (11–26)	14 (6–24)	<0.001
After change in guidelines	3 (2–4)	9 (6–11)	9 (6–13)	11 (9–19)	13 (7–19)	<0.001

ALC, absolute lymphocyte count; IQR, interquartile range.

a Patients who did not have chest radiographs available for review: missing chest x-ray plate, chest x-ray not performed on admission, or chest x-ray not performed during the course of admission.

b Ground glass opacity in chest radiographs was defined as haziness of the lung parenchyma with preservation of the bronchovascular margins (Hansell et al., 2008). Cochran–Armitage test for trend for categorical variables, and Jonckheere–Terpstra trend test for continuous variables.

c Decreased sensorium or impaired consciousness : confused, disoriented, somnolent, lethargic, obtunded, stuporous, comatosed

d Before the adoption of the symptom-based isolation strategy

All asymptomatic patients underwent at least one laboratory test, while patients with severe or critical disease underwent extensive diagnostic evaluation (Table S2, see online supplementary material). Abnormal laboratory results were observed with increasing severity of disease (Table 3; also see Table S3 in the online supplementary material for the limits of normal haematologic and blood chemistries for adults). The majority (*n*=1424, 94.9%) of patients had a chest x-ray. More severe and critical cases had ground glass opacities, consolidation, and infiltrates affecting >50% of the lungs. Pleural effusion (6.7%) and pneumothorax (0.3%) were uncommon (see Table S1 in the online supplementary material for detailed radiographic findings).

Pairwise analysis did not show significant differences between mild and asymptomatic patients in terms of age, vital signs on admission and multiple laboratory parameters (Table S4, see online supplementary material).

### Outcomes

**Figure 3:**
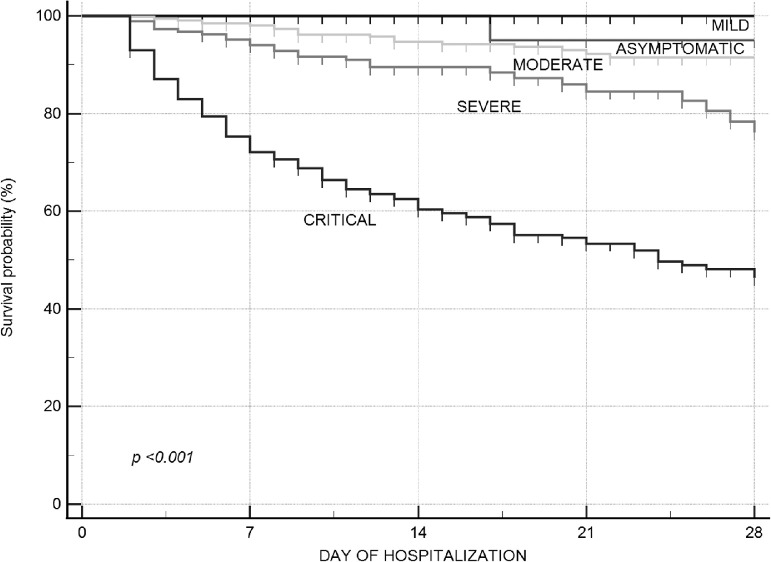
Kaplan–Meier survival curve with log-rank test of 28-day mortality among 1500 patients with confirmed coronavirus disease 2019 admitted to the University of the Philippines–Philippine General Hospital, based on severity of disease. The corresponding severity of disease is indicated below the curve/line.

The overall mortality rate was 15.1% (*n*=226). Greater severity of disease was associated with mortality (Table 3), as shown in the Kaplan–Meier survival curve (Figure 3). One asymptomatic patient died of nosocomial pneumonia after developing profound neutropenia following chemotherapy for acute leukaemia.

The overall median length of hospitalization was 12 (IQR 7–19) days, and was shorter for mild cases (Table 3). Change in the discharge guidelines resulted in a significant reduction (*P*<0.05) in the overall length of hospitalization from a median of 13 (IQR 7–21) days to 9 (IQR 5–14) days. This was observed across all patient groups.

## Discussion

This study validates published findings that have reported the following: (1) association between COVID-19, age and co-morbidities (Jin et al., 2020; Salva et al., 2020; Abad et al., 2021; Mishra et al., 2020; Salamat et al., 2021); (2) common clinical presentations including cough, fever and shortness of breath (Cao et al., 2020; Jin et al., 2020; Jutzeler et al., 2020; Salva et al., 2020; Wong et al., 2020; Abad et al., 2021; Salamat et al., 2021); and (3) overall mortality rate of 15% (Salva et al., 2020; Abad et al., 2021). In addition, this study highlights other important insights about COVID-19: (1) asymptomatic disease occurs frequently (14.8%) and is captured on routine evaluation; (2) COVID-19 has a bimodal age distribution; (3) HCWs are at high risk of COVID-19; and (4) a symptom-based strategy reduces the duration of hospitalization considerably.

COVID-19 in the Philippines has been reported to affect older age groups disproportionately (Haw et al., 2020). However, the findings of the present study showed a bimodal distribution of cases (Figure 2) with two distinct populations: a young, economically productive population and an older population. Studies have consistently shown old age as a significant risk factor for infection, hospitalization and adverse outcomes (Wu et al., 2020; Zhou et al., 2020; Garibaldi et al., 2021), presumably due to immunosenescence and increased prevalence of co-morbidities (Perrotta et al., 2020; Bajaj et al., 2021; Chen et al., 2021). In the USA, hospitalization is almost twice as long for patients aged 65–74 years compared with those aged 50–64 years, and five times as long compared with patients aged 18–49 years (CDC COVID-19 Response Team, 2020; Garg et al., 2020). Quarantine restrictions in Manila preclude the elderly from leaving the household, mitigating their risk of acquiring COVID-19. However, if members of the same household frequently come and go, this may explain disease transmission and subsequent infection of the elderly. A meta-analysis reported an estimated household secondary attack rate from both asymptomatic [0.7%; 95% confidence interval (CI) 0–4.9%] and symptomatic (18.0%; 95% CI 14.2–22.1%) individuals (Madewell et al., 2020). It is hypothesized that the bimodal distribution may be indirect evidence of transmission of SARS-CoV-2 within households, where asymptomatic young adults drive community transmission and infect the elderly in the household.

The present findings also reflect global trends of increasing infection among younger, economically productive age groups (Salvatore et al., 2020). This can be attributed to higher exposure related to the resumption of activities in schools and workplaces, non-compliance with restrictions and/or the presence of co-morbidities (Czeisler et al., 2020; Salvatore et al., 2020). Since HCWs and pregnant patients comprised almost one-quarter of the study population (Table 2), the data are skewed, leading to a younger cohort of patients.

It is possible that many infections among the HCWs in this study were detected because of better access to testing, lower threshold for SARS-CoV-2 testing, and implementation of hospital-wide surveillance for COVID-19. Nonetheless, studies have documented a higher risk of COVID-19 among HCWs, with reported prevalence of 11% (95% CI 7–15%) (Gómez-Ochoa et al., 2021). Poor hand hygiene, inadequate access to and utilization of personal protective equipment (PPE), and exposure to aerosol-generating procedures have been identified as risk factors for COVID-19 in the healthcare setting (El‐Boghdadly et al., 2020; Ran et al., 2020; Wang et al., 2020). HCWs comprised 20.3% of the cohort in the present study. The authors were unable to determine whether the infection resulted from nosocomial or community-acquired transmission. However, genomic studies have suggested that community-acquired COVID-19 introduced to the healthcare setting is a more common occurrence than hospital-acquired infection (Sikkema et al., 2020). Further studies on this aspect are recommended given its implications for infection control.

On the other hand, the high detection rate of COVID-19 among pregnant patients may be related to frequent prenatal check-ups and universal testing for SARS-CoV-2 at UP-PGH. COVID-19 is a concern among pregnant women given the higher risk for postpartum complications including haemorrhage, admission to the intensive care unit, and intrauterine fetal demise (Hcini et al., 2021). Feto-maternal complications from COVID-19 are beyond the scope of this study, and further studies on this subgroup of patients are recommended.

Fifteen percent of the study cohort had asymptomatic COVID-19. Despite the absence of symptoms, many still had laboratory tests performed. Not surprisingly, the majority of these blood tests were within normal limits. From a cost-effectiveness standpoint, especially in resource-limited settings, laboratory work-up for these patients is likely unnecessary and should be done on a case-by-case basis. On the other hand, chest imaging may be warranted as some individuals thought to be asymptomatic were reclassified after radiological assessment. However, as per national recommendations, asymptomatic cases and patients with mild COVID-19 can be monitored in designated community care and quarantine facilities to ensure that patients with more severe disease will have access to hospital services (Philippine Society for Microbiology and Infectious Diseases et al., 2020).

National and hospital policies changed periodically during the study period in response to the evolving pandemic. The implementation of universal testing for COVID-19 at UP-PGH has been critical in the identification of patients with subclinical COVID-19. Early identification of these patients has important clinical implications in terms of preventing nosocomial transmission through isolation of infected patients, and monitoring disease progression to initiate timely therapeutic interventions. Several other hospitals have also implemented universal testing, with positivity rates ranging from 0.03% to 4.5% (Sastry et al., 2020; Nakamura and Itoi, 2021; Saidel-Odes et al., 2021). Positivity rates were influenced by local prevalence and the level of community transmission. Data on the cost-effectiveness of universal screening are limited. However, in areas where the prevalence rate and level of community transmission are high [i.e. ≥100 new cases per 100,000 persons in the past 7 days, or ≥10% positive nucleic acid amplification tests during the past 3 days (Centers for Disease Control and Prevention, 2021)], this may be an acceptable strategy to guide bed allocation and use of PPE, and to prevent unnecessary quarantine of exposed HCWs and nosocomial transmission.

Changes in the local guidelines affected the length of hospitalization of patients with COVID-19 significantly at UP-PGH. The implementation of a symptom-based strategy resulted in a shorter duration of hospitalization, especially for patients with mild disease. A US study showed similar results with excess length of stay for acute care as well as extra cost for test-based vs symptom-based isolation strategies (Wu et al., 2021). Studies have shown that RT-PCR positivity for SARS-CoV-2 may persist beyond infectivity, and that viable SARS-CoV-2 was not isolated among patients beyond 8 days since symptom onset (Bullard et al., 2020; Xiao et al., 2020). The shorter length of stay had several positive consequences, including allocation of badly needed beds, and decreased cost for those who were admitted. However, the need to reallocate hospital beds must be balanced with the possibility of disease transmission from individuals recovering from COVID-19. Clinical data for patients with severe or critical disease or immunocompromised patients are limited, and viral shedding can theoretically be longer. As such, isolation was extended to 21 days for this subpopulation of patients (Philippine Society for Microbiology and Infectious Diseases et al., 2020). As more is learned about the mechanics of transmission of the variants of concern, guidance regarding symptom-based isolation protocols may also evolve.

### Implications for clinical practice

The economic impact and healthcare burden of COVID-19 necessitates evidence-based changes in policies to mitigate these problems. The study findings show that: (1) blood tests among asymptomatic cases and patients with mild COVID-19 are likely to be unnecessary; (2) universal testing in areas with a high level of community transmission can guide resource and bed allocation; and (3) a symptom-based isolation protocol can address issues of limited bed capacity.

### Study limitations

This study has limitations inherent to its retrospective nature, including the possibility of information and misclassification bias. However, any information bias from missing data was mitigated by the use of a hospital clinical pathway for patients with COVID-19 which guided systematic recording of clinical information. Misclassification bias could have resulted if asymptomatic patients were found to have pneumonia on chest x-ray (i.e. 30% of asymptomatic patients did not have imaging, and may have been misclassified as asymptomatic rather than moderate cases, underestimating the proportion of patients with moderate disease). National guidelines were used for the classification of COVID-19 severity, but these are comparable with international definitions. The authors were unable to identify presymptomatic patients as asymptomatic patients were not followed-up to assess if they developed symptoms within 14 days of a positive SARS-CoV-2 test result. In addition, the authors were unable to perform genomic analysis, which could have helped evaluate transmission dynamics. These findings may not be generalizable due to differences between populations, differences in hospital policies, differences in clinical practice, and differences in the level of community transmission. Despite these limitations, however, this study has several strengths. A large cohort of consecutive patients was assessed, including a good proportion of patients with asymptomatic disease (this group is often excluded from analysis of hospitalized patients). In addition, the authors were able to discuss changes in hospital policy over the study period which impacted infection control strategies and patient outcomes.

## Conclusion

The clinical profile of patients with COVID-19 in this study was consistent with published reports globally. Asymptomatic cases and patients with mild COVID-19 share similar clinical features, while markers of inflammation increase and signs of organ dysfunction become more evident in patients with severe and critical disease. Asymptomatic COVID-19 is common, and blood tests are likely to be unnecessary. Universal testing may be a valuable strategy in areas of high community transmission, given its implications for infection control. The overall mortality rate of COVID-19 was high at 15.1%. The symptom-based strategy shortened the length of hospitalization and should be recommended.

## Appendix Supplementary materials

Image, application 1
