# Supplementary material for: Clinical characteristics of patients with asymptomatic and symptomatic COVID-19 admitted to a tertiary referral centre in the Philippines
Source: IJID Reg. 2022 Feb 6;2:204–11. doi: 10.1016/j.ijregi.2022.02.002 (PMC8818128; doi:10.1016/j.ijregi.2022.02.002)
Supplement: Supplementary file 1 [file mmc1.docx]

**SUPPLEMENTARY MATERIALS**

**Supplementary Table 1.** Clinical characteristics on admission and outcomes of patients with COVID-19 infection in UP-PGH (full list)

**Supplementary Table 2.** Number and percentage of COVID-19 infected patients who underwent diagnostic tests, according to spectrum of disease activity

**Supplementary Table 3.** Limits of Normal Hematologic and Blood Chemistries for Adults

**Supplementary Table 4.** Conover’s test of multiple pairwise comparisons following a statistically significant (p<0.05) Jonckheere-Terpstra trend test for continuous variables.

**Supplementary Table 1.** Clinical characteristics on admission and outcomes of patients with COVID-19 infection in UP-PGH (full list)

**DISEASE ACTIVITY ON ADMISSION**

**Overall Asymptomatic Mild Moderate Severe Critical P value**

**(N=1500) (N=222) (N=203) (N=549) (N=185) (N=341)**

**SYMPTOMS**, No. (%)

Headache 101(6.7) - 27 (13.3) 50 (9.1) 12 (6.5) 12 (3.5) 0.650

Chills 51 (3.4) - 11 (5.4) 23 (4.2) 2 (1.1) 15 (4.4) 0.144

Fever 703 (46.9) - 97 (47.8) 269 (49.0) 108 (58.4) 229 (67.2) <0.001

Cough 752 (50.1) - 91 (44.8) 285 (51.9) 132 (71.4) 244 (71.6) <0.001

Rhinorrhea / Congestion 163 (10.9) - 49 (24.1) 68 (12.4) 19 (10.3) 27 (7.9) 0.995

Shortness of Breath 558 (37.2) - 40 (19.7) 145 (26.4) 130 (70.3) 243 (71.3) <0.001

Sore throat 183 (12.2) - 61 (30.0) 72 (13.1) 14 (7.6) 36 (10.6) 0.912

Myalgia 94 (6.3) - 26 (12.8) 46 (8.4) 10 (5.4) 12 (3.5) 0.640

Malaise / Fatigue /

Generalized Weakness 345 (23.0) - 50 (24.6) 132 (24.0) 58 (31.4) 105 (30.8) <0.001

Diarrhea 199 (13.3) - 44 (21.7) 76 (13.8) 27 (14.6) 52 (15.2) <0.001

Nausea or Vomiting 65 (4.3) - 7 (3.4) 23 (4.2) 14 (7.6) 21 (6.2) <0.001

Decreased Appetite 161 (10.7) - 9 (4.4) 44 (8.0) 33 (17.8) 75 (22.0) <0.001

Abdominal pain / discomfort 58 (3.9) - 9 (4.4) 26 (4.7) 9 (4.9) 14 (4.1) 0.048

Change or Loss in Taste 94 (6.3) - 17 (8.4) 39 (7.1) 14 (7.6) 24 (7.0) 0.012

Change or Loss in Smell 94 (6.3) - 34 (16.7) 39 (7.1) 7 (3.8) 14 (4.1) 0.302

Decreased Sensorium 88 (5.9) - 5 (2.5) 23 (4.2) 7 (3.8) 53 (15.5) <0.001

**VITAL SIGNS**, median (IQR)

Systolic blood pressure, mmHg 125 (115 - 140) 120 (110 - 130) 120 (110 - 130) 130 (120 - 140) 130 (120 - 144) 130 (113 - 145) <0.001

Diastolic blood pressure, mmHg 80 (70 - 82) 80 (70 - 80) 78 (70 - 80) 80 (70 - 86) 80 (70 - 85) 80 (70 - 85) 0.069

Mean arterial pressure, mmHg 93 (87 - 103) 93 (83 - 97) 92 (83 - 100) 93 (87 - 103) 97 (90 - 103) 96 (86 - 103) <0.001

Heart rate, beats/min 87 (78 - 98) 85 (78 - 92) 83 (76 - 91) 84 (78 - 92) 90 (80 - 102) 98 (85 - 112) <0.001

Respiratory rate, breaths/min 20 (20 - 23) 20 (19 - 20) 20 (18 - 20) 20 (20 - 20) 22 (20 - 24) 26 (23 - 30) <0.001

Temperature, ^o^C 36.6 (36.3 - 37.0) 36.6 (36.4 - 36.9) 36.5 (36.1 - 36.8) 36.6 (36.2 - 37.0) 36.7 (36.5 - 37.3) 36.7 (36.3 - 37.0) <0.001

Peripheral O_2_ saturation, % 97 (95 - 98) 98 (97 - 99) 98 (97 - 99) 98 (96 - 98) 95 (91 - 97) 92 (81 - 96) <0.001

Glasgow Coma Scale score 15 (15 - 15) 15 (15 - 15) 15 (15 - 15) 15 (15 - 15) 15 (15 - 15) 15 (15 - 15) <0.001

**LABORATORY FINDINGS**

***Complete Blood Count***, median (IQR)

Hemoglobin, g/L 130 (116 - 143) 127 (117 - 137) 140 (132 - 149) 130 (114 - 143) 124 (105 - 138) 128 (110 - 143) <0.001

Hematocrit, 40.0 (35.0 - 43.0) 39.0 (35.0 - 42.0) 42.0 (40.0 - 44.5) 39.0 (34.0 - 43.0) 37.0 (32.0 - 42.0) 39.0 (34.0 - 43.0) 0.002

White blood cells, x10^9^/L 8.2 (6.0 - 11.3) 10.3 (8.0 - 12.7) 7.1 (5.2 - 8.9) 7.2 (5.5 - 9.4) 7.8 (5.8 - 10.3) 10.6 (7.4 - 14.9) <0.001

Neutrophil, % 70.0 (60.0 - 81.0) 70.0 (63.3 - 77.0) 59.0 (51.0 - 66.0) 65.0 (56.0 - 74.0) 76.0 (69.0 - 83.0) 84.0 (76.0 - 89.0) <0.001

Lymphocyte, % 19.0 (10.0 - 27.3) 20.0 (16.0 - 26.0) 29.0 (23.0 - 36.0) 23.0 (14.0 - 31.0) 14.0 (9.0 - 20.0) 8.0 (5.0 - 15.0) <0.001

ALC, x 10^9^/L 1.46 (0.96 - 2.03) 1.97 (1.57 - 2.48) 1.96(1.56 - 2.44) 1.53(1.09 - 2.06) 1.00(0.80 - 1.35) 0.91 (0.58 - 1.39) <0.001

Neutrophil lymphocyte ratio 3.7 (2.2 - 7.6) 3.5 (2.4 - 4.9) 2.0 (1.5 - 2.8) 2.8 (1.8 - 5.2) 5.5 (3.5 - 8.6) 9.8 (5.1 - 17.6) <0.001

Platelet, x10^9^/L 278 (209 - 356) 284 (237 - 347) 293 (238 - 336) 271 (202 - 364) 270(181 - 385) 267 (203 - 343) 0.009

***Arterial blood gas***, median (IQR)

pH 7.42 (7.39 - 7.45) 7.42 (7.40 - 7.44) 7.41 (7.39 - 7.43) 7.43 (7.40 - 7.46) 7.43 (7.40 - 7.46) 7.42 (7.36 - 7.46) 0.957

pCO_2_ 35.0 (29.8 - 39.0) 39.0 (35.8 - 42.3) 38.0 (34.0 - 41.0) 35.0 (30.4 - 39.0) 33.0 (28.0 - 38.0) 32.0 (27.0 - 37.0) <0.001

pO_2_ 90.0 (77.0 - 107.0) 98.0 (89.8 - 107.3) 98.1 (90.0 - 111.0) 88.0 (78.0 - 99.0) 102.4(74.0 - 140.2)81.0 (63.0 - 111.3) <0.001

HCO_3_ 23.1 (19.1 - 25.9) 25.7 (22.9 - 27.2) 24.2 (22.3 - 25.9) 23.6 (20.3 - 26.0) 22.9 (18.5 - 26.1) 19.5 (16.6 - 23.6) <0.001

O_2_ saturation 97.0 (95.3 - 98.0) 98.0 (97.0 - 98.0) 98.0 (97.0 - 98.0) 97.0 (96.0 - 98.0) 98.0 (95.3 - 99.0) 96.0 (91.2 - 98.0) <0.001

PO_2_ and FiO_2_ ratio 386 (256 - 462) 467 (424 - 511) 467 (429 - 519) 410 (360 - 462) 338 (281 - 425) 175 (108 - 262) <0.001

***Blood Chemistry***, median (IQR)

Blood urea nitrogen, mmol/L 4.8 (3.5 - 8.1) 3.3 (2.5 - 4.4) 3.8 (3.1 - 4.6) 4.6 (3.3 - 7.1) 5.8 (4.3 - 11.1) 7.8 (5.0 - 15.3) <0.001

Serum creatinine, µmol/L 72.0 (55.0 - 103.0) 56.0 (46.0 - 74.5) 61.5 (52.0 - 76.5) 70.0 (54.0 - 97.0) 80.0 (60.0 - 127.3) 93.0 (69.0 - 165.5) <0.001

eGFR^a^ mL/min/1.73m^2^ 94 (61 - 113) 119 (103 - 128) 116 (102 - 125) 92 (63 - 110) 87 (46 - 105) 69 (33 - 94) <0.001

Aspartate aminotransferase, U/L 43.0 (31.0 - 68.0) 31.0 (25.0 - 37.0) 32.0 (26.0 - 44.0) 40.0 (30.0 - 61.0) 55.0 (40.0 - 81.3) 64.0 (48.0 - 97.0) <0.001

Alanine aminotransferase, IU/L 34.0 (19.0 - 65.0) 18.0 (12.0 - 34.0) 30.0 (17.0 - 54.5) 31.5 (21.0 - 67.0) 45.0 (21.0 - 80.0) 46.0 (26.0 - 79.3) <0.001

Albumin, g/L 37.0 (33.0 - 42.0) 39.0 (35.8 - 44.0) 45.0 (41.0 - 47.0) 38.0 (34.0 - 43.0) 35.0 (32.0 - 38.0) 34.0 (30.0 - 38.0) <0.001

Total bilirubin, mg/dl 0.67 (0.50 - 0.98) 0.57 (0.42 - 0.87) 0.55 (0.44 - 0.77) 0.63 (0.49 - 0.89) 0.76 (0.49 - 0.99) 0.86 (0.58 - 1.20) <0.001

Direct bilirubin, mg/dl 0.27 (0.19 - 0.43) 0.19 (0.11 - 0.23) 0.20 (0.14 - 0.25) 0.25 (0.17 - 0.37) 0.33 (0.23 - 0.47) 0.42 (0.30 - 0.64) <0.001

Indirect bilirubin, mg/dl 0.38 (0.22 - 0.61) 0.42 (0.21 - 0.74) 0.37 (0.23 - 0.53) 0.37 (0.22 - 0.61) 0.37 (0.18 - 0.55) 0.42 (0.23 - 0.64) 0.664

***Inflammatory Markers***, median (IQR)

Lactate dehydrogenase, U/L 301 (232 - 455) 233 (203 - 279) 220 (187 - 253) 281 (230 - 353) 374 (307 - 487) 541 (390 - 748) <0.001

Serum ferritin, ng/mL 481 (170 - 1218) 89 (38 - 229) 126 (56 - 330) 407 (196 - 806) 1000 (460 - 1950) 1280 (704 - 2280) <0.001

Serum procalcitonin, ng/mL 0.13 (0.04 - 0.55) 0.07 (0.04 - 0.11) 0.04 (0.04 - 0.05) 0.07 (0.04 - 0.28) 0.24 (0.09 - 1.04) 0.47 (0.17 - 1.65) <0.001

D-dimer, ug/mL 1.28 (0.56 - 3.06) 0.62 (0.32 - 1.42) 0.40 (0.30 - 0.74) 0.82 (0.42 - 1.86) 1.68 (0.87 - 3.23) 2.74 (1.44 - 7.52) <0.001

C-reactive protein, No. (%)

No CRP test done 322 (21.5) 129 (58.1) 31 (15.3) 109 (19.9) 23 (12.4) 30 (8.8)

≤12 mg/L 497 (33.1) 84 (37.8) 143 (70.4) 221 (40.3) 25 (13.5) 24 (7.0)

>12 mg/L 681 (45.4) 9 (4.1) 29 (14.3) 219 (39.9) 137 (74.1) 287 (84.2) <0.001

**CHEST RADIOGRAPH,** No. (%)

No chest xray^b^ 76 (5.1) 69 (31.1) 3 (1.5) 2 (0.4) 1 (0.5) 1 (0.3)

*Pulmonary Infiltrates*

Bilateral 727 (48.5) 0 (0) 0 (0) 261 (47.5) 160 (86.5) 306 (89.7) <0.001

More than 50% of the lungs 499 (33.3) 0 (0) 0 (0) 109 (19.9) 129 (69.7) 261 (76.5) <0.001

Limited - periphery 85 (5.7) 0 (0) 0 (0) 52 (9.5) 16 (8.6) 17 (5.0) 0.011

*Density*

Ground glass^c^ 647 (43.1) 0 (0) 0 (0) 244 (44.4) 134 (72.4) 269 (78.9) <0.001

Consolidation 103 (6.9) 0 (0) 0 (0) 13 (2.4) 22 (11.9) 68 (19.9) <0.001

Nodular 72 (4.8) 0 (0) 0 (0) 36 (6.6) 13 (7.0) 23 (6.7) <0.001

Reticular 225 (15.0) 0 (0) 0 (0) 150 (27.3) 35 (18.9) 40 (11.7) <0.001

*Other Findings*

Pleural effusion 100 (6.7) 1 (0.5) 2 (1.0) 35 (6.4) 29 (15.7) 33 (9.7) <0.001

Pneumothorax 5 (0.3) 0 (0) 0 (0) 0 (0) 1 (0.5) 4 (1.2) 0.006

**MORTALITY**

Number (%) 226 (15.1) 1 (0.5) 0 (0) 34 (6.2) 29 (15.7) 162 (47.5) <0.001

**LENGTH OF HOSPITAL STAY**

Days, Median (IQR) 12 (7 - 19) 4 (3 - 9) 11 (7 - 14) 12 (8 - 20) 15 (10 - 24) 14 (7 - 21) <0.001

Before change in guidelines, days 13 (7 - 21) 7 (4 - 12) 12 (7 - 17) 13 (9 - 23) 16 (11 - 26) 14 (6 - 24) <0.001

After change in guidelines, days 9 (5 - 14) 3 (2 - 4) 9 (6 - 11) 9 (6 - 13) 11 (9 - 19) 13 (7 - 19) <0.001

a. eGFR or estimated glomerular filtration rate calculated using the Chronic Kidney Disease Epidemiology Collaboration (CKD-EPI) equation

b. Patients who did not have chest radiographs available for review: missing chest xray plate, chest xray not done initially on admission, or chest xray not done during the course of admission

c. Ground glass opacity in chest radiographs was defined as haziness of the lung parenchyma with preservation of the bronchovascular margins(Hansell et al., 2008)

Cochran-Armitage test for trend for categorical variables, and the Jonckheere-Terpstra trend test for continuous variables

ALC – Absolute Lymphocyte Count

**Supplementary Table 2.** Number and percentage of COVID-19 infected patients who underwent diagnostic tests, according to disease activity

**DISEASE ACTIVITY ON ADMISSION**

**DIAGNOSTIC TESTS OVERALL ASYMPTOMATIC MILD MODERATE SEVERE CRITICAL**

**(N=500) (N=222) (N=203) (N=549) (N=185) (N=341)**

n (%) n (%) n (%) n (%) n (%) n (%)

Complete Blood Count 1440 (96.0) 207 (93.2) 197 (97.0) 527 (96.0) 174 (94.1) 335 (98.2)

Arterial Blood Gas 1218 (81.2) 69 (31.1) 179 (88.2) 460 (83.8) 173 (93.5) 337 (98.8)

Blood Urea Nitrogen 1292 (86.1) 135 (60.8) 186 (91.6) 481 (87.6) 167 (90.3) 323 (94.7)

Serum creatinine 1350 (90.0) 149 (67.1) 188 (92.6) 508 (92.5) 173 (93.5) 332 (97.4)

Aspartate aminotransferase 1291 (86.1) 140 (63.1) 185 (91.1) 485 (88.3) 161 (87.0) 320 (93.8)

Alanine aminotransferase 1297 (86.5) 140 (63.1) 184 (90.6) 490 (89.3) 162 (87.6) 321 (94.1)

Albumin 1100 (73.3) 105 (47.3) 129 (63.5) 406 (74.0) 155 (83.8) 305 (89.4)

Bilirubins 992 (66.1) 59 (26.6) 135 (66.5) 375 (68.3) 144 (77.8) 279 (81.8)

Lactate dehydrogenase 1295 (86.3) 135 (60.8) 185 (91.1) 493 (89.8) 162 (87.6) 320 (93.8)

Serum ferritin 1291 (86.1) 120 (54.1) 194 (95.6) 491 (89.4) 162 (87.6) 324 (95.0)

C-reactive protein 1178 (78.5) 93 (41.9) 172 (84.7) 440 (80.1) 162 (87.6) 311 (91.2)

Serum procalcitonin 727 (48.5) 43 (19.4) 82 (40.4) 275 (50.1) 104 (56.2) 223 (65.4)

D-dimer 843 (56.2) 46 (20.7) 96 (47.3) 299 (54.5) 134 (72.4) 268 (78.6)

Chest x-ray 1424 (94.9) 153 (68.9) 200 (98.5) 547 (99.6) 184 (99.5) 340 (99.7)

**Supplementary Table 3.** Limits of Normal Hematologic and Blood Chemistries for Adults

***Complete Blood Count***,

Hemoglobin, g/L M: 13.5 – 17.5, F: 12.0 – 16.0

Hematocrit, M: 42 – 54, F: 37 - 47

White blood cells, x10^9^/L 4.0 – 10.0

Neutrophil, % 46 – 78

Lymphocyte, % 18 – 52

Platelet, x10^9^/L 11.5 – 14.5

***Arterial blood gas***

pH 7.35 – 7.45

pCO_2_ M: 35 – 48, F: 32 – 45

pO_2_ 83 – 108

HCO_3_ 18 – 23

O_2_ saturation, % 95 – 98

***Blood Chemistry***

Blood urea nitrogen, mmol/L 2.86 – 7.5

Serum creatinine, µmol/L M: 66.3 – 106.1, F: 57.5 – 88.4

Aspartate aminotransferase, U/L 3 – 44

Alanine aminotransferase, IU/L 0 – 40

Albumin, g/L 35.0 – 50.0

Total bilirubin, mg/dl 0.2 – 1.3

Direct bilirubin, mg/dl 0.27 (0.19 - 0.43)

Indirect bilirubin, mg/dl 0.38 (0.22 - 0.61)

Reference: Rush University (2021, December) Normal ranges for common laboratory tests. Retrieved 28 December 2021, from <https://rml.rush.edu/Pages/RMLRanges.aspx>.

**Supplementary Table 4**. Conover’s test of multiple pairwise comparisons following a statistically significant (p<0.05) Jonckheere-Terpstra trend test for continuous variables.

**VARIABLE ILLNESS SEVERITY ON ADMISSION AVERAGE RANK DIFFERENT (p<0.05)**

Age Asymptomatic 360.8 (Moderate)(Severe)(Critical)

Mild 355.56 (Moderate)(Severe)(Critical)

Moderate 822.82 (Asymptomatic)(Mild)(Severe)(Critical)

Severe 915.65 (Asymptomatic)(Mild)(Moderate)(Critical)

Critical 1033.28 (Asymptomatic)(Mild)(Moderate)(Severe)

***Vital Signs on Admission***

Systolic blood pressure Asymptomatic 603.37 (Moderate)(Severe)(Critical)

Mild 624.19 (Moderate)(Severe)(Critical)

Moderate 806.43 (Asymptomatic)(Mild)

Severe 844.21 (Asymptomatic)(Mild)

Critical 780.59 (Asymptomatic)(Mild)

Mean arterial pressure Asymptomatic 636.61 (Moderate)(Severe)(Critical)

Mild 652.79 (Moderate)(Severe)(Critical)

Moderate 792.07 (Asymptomatic)(Mild)

Severe 837.93 (Asymptomatic)(Mild)

Critical 768.45 (Asymptomatic)(Mild)

Heart rate Asymptomatic 670.1 (Severe)(Critical)

Mild 628.91 (Severe)(Critical)

Moderate 666.92 (Severe)(Critical)

Severe 825.44 (Asymptomatic)(Mild)(Moderate)(Critical)

Critical 969.13 (Asymptomatic)(Mild)(Moderate)(Severe)

Respiratory rate Asymptomatic 492.3 (Moderate)(Severe)(Critical)

Mild 493.26 (Moderate)(Severe)(Critical)

Moderate 606.06 (Asymptomatic)(Mild)(Severe)(Critical)

Severe 956.49 (Asymptomatic)(Mild)(Moderate)(Critical)

Critical 1192.52 (Asymptomatic)(Mild)(Moderate)(Severe)

Temperature Asymptomatic 725.28 (Mild)(Severe)(Critical)

Mild 587.89 (Asymptomatic)(Moderate)(Severe)(Critical)

Moderate 719.59 (Mild)(Severe)(Critical)

Severe 856.11 (Asymptomatic)(Mild)(Moderate)

Critical 799.5 (Asymptomatic)(Mild)(Moderate)

Peripheral O_2_ saturation Asymptomatic 917.9 (Moderate)(Severe)(Critical)

Mild 971.57 (Moderate)(Severe)(Critical)

Moderate 828.74 (Asymptomatic)(Mild)(Severe)(Critical)

Severe 525.04 (Asymptomatic)(Mild)(Moderate)(Critical)

Critical 409.14 (Asymptomatic)(Mild)(Moderate)(Severe)

Glasgow Coma Scale score Asymptomatic 791.77 (Critical)

Mild 791.11 (Critical)

Moderate 786.48 (Critical)

Severe 794.66 (Critical)

Critical 617.57 (Asymptomatic)(Mild)(Moderate)(Severe)

***Complete Blood Count***

Hemoglobin Asymptomatic 673.4 (Mild)

Mild 947.41 (Asymptomatic)(Moderate)(Severe)(Critical)

Moderate 714.5 (Mild)(Severe)

Severe 603.6 (Mild)(Moderate)(Critical)

Critical 686.29 (Mild)(Severe)

Hematocrit Asymptomatic 680.74 (Mild)

Mild 933.81 (Asymptomatic)(Moderate)(Severe)(Critical)

Moderate 700.55 (Mild)(Severe)

Severe 603.64 (Mild)(Moderate)(Critical)

Critical 705.8 (Mild)(Severe)

White blood cells Asymptomatic 917.79 (Mild)(Moderate)(Severe)

Mild 555.85 (Asymptomatic)(Severe)(Critical)

Moderate 599.08 (Asymptomatic)(Critical)

Severe 659.25 (Asymptomatic)(Mild)(Critical)

Critical 918.24 (Mild)(Moderate)(Severe)

Neutrophil Asymptomatic 798.05 (Mild)(Severe)(Critical)

Mild 1058.62 (Asymptomatic)(Moderate)(Severe)(Critical)

Moderate 843.27 (Mild)(Severe)(Critical)

Severe 546.1 (Asymptomatic)(Mild)(Moderate)(Critical)

Critical 364.29 (Asymptomatic)(Mild)(Moderate)(Severe)

Lymphocyte Asymptomatic 798.05 (Mild)(Severe)(Critical)

Mild 1058.62 (Asymptomatic)(Moderate)(Severe)(Critical)

Moderate 843.27 (Mild)(Severe)(Critical)

Severe 546.1 (Asymptomatic)(Mild)(Moderate)(Critical)

Critical 364.29 (Asymptomatic)(Mild)(Moderate)(Severe)

Absolute lymphocyte count Asymptomatic 997.19 (Moderate)(Severe)(Critical)

Mild 989.59 (Moderate)(Severe)(Critical)

Moderate 768.1 (Asymptomatic)(Mild)(Severe)(Critical)

Severe 468.38 (Asymptomatic)(Mild)(Moderate)

Critical 441.06 (Asymptomatic)(Mild)(Moderate)

Neutrophil lymphocyte ratio Asymptomatic 654.28 (Mild)(Moderate)(Severe)(Critical)

Mild 379.3 (Asymptomatic)(Moderate)(Severe)(Critical)

Moderate 588.48 (Asymptomatic)(Mild)(Severe)(Critical)

Severe 885.9 (Asymptomatic)(Mild)(Moderate)(Critical)

Critical 1075.34 (Asymptomatic)(Mild)(Moderate)(Severe)

***Arterial blood gas***

pCO_2_ Asymptomatic 833.86 (Moderate)(Severe)(Critical)

Mild 763.35 (Moderate)(Severe)(Critical)

Moderate 621.3 (Asymptomatic)(Mild)(Severe)(Critical)

Severe 539.62 (Asymptomatic)(Mild)(Moderate)

Critical 499.84 (Asymptomatic)(Mild)(Moderate)

pO_2_ Asymptomatic 716.36 (Moderate)(Critical)

Mild 766.2 (Moderate)(Severe)(Critical)

Moderate 561.48 (Asymptomatic)(Mild)(Severe)

Severe 693.14 (Mild)(Moderate)(Critical)

Critical 527 (Asymptomatic)(Mild)(Severe)

HCO_3_ Asymptomatic 831.61 (Mild)(Moderate)(Severe)(Critical)

Mild 724.26 (Asymptomatic)(Moderate)(Severe)(Critical)

Moderate 657.3 (Asymptomatic)(Mild)(Critical)

Severe 599.77 (Asymptomatic)(Mild)(Critical)

Critical 442.81 (Asymptomatic)(Mild)(Moderate)(Severe)

O_2_ saturation Asymptomatic 708.96 (Moderate)(Critical)

Mild 745.1 (Moderate)(Critical)

Moderate 572.71 (Asymptomatic)(Mild)(Severe)(Critical)

Severe 694.52 (Moderate)(Critical)

Critical 515.61 (Asymptomatic)(Mild)(Moderate)(Severe)

pO_2_ and FiO_2_ ratio Asymptomatic 874.55 (Moderate)(Severe)(Critical)

Mild 915.37 (Moderate)(Severe)(Critical)

Moderate 720.62 (Asymptomatic)(Mild)(Severe)(Critical)

Severe 567.77 (Asymptomatic)(Mild)(Moderate)(Critical)

Critical 262.51 (Asymptomatic)(Mild)(Moderate)(Severe)

***Blood Chemistry***

Blood urea nitrogen Asymptomatic 368.54 (Moderate)(Severe)(Critical)

Mild 434.83 (Moderate)(Severe)(Critical)

Moderate 611.81 (Asymptomatic)(Mild)(Severe)(Critical)

Severe 766.38 (Asymptomatic)(Mild)(Moderate)(Critical)

Critical 874.24 (Asymptomatic)(Mild)(Moderate)(Severe)

Serum creatinine Asymptomatic 431.39 (Mild)(Moderate)(Severe)(Critical)

Mild 515.83 (Asymptomatic)(Moderate)(Severe)(Critical)

Moderate 659.84 (Asymptomatic)(Mild)(Severe)(Critical)

Severe 758.88 (Asymptomatic)(Mild)(Moderate)(Critical)

Critical 855.98 (Asymptomatic)(Mild)(Moderate)(Severe)

eGFR Asymptomatic 987.76 (Moderate)(Severe)(Critical)

Mild 967.68 (Moderate)(Severe)(Critical)

Moderate 655.63 (Asymptomatic)(Mild)(Severe)(Critical)

Severe 567.98 (Asymptomatic)(Mild)(Moderate)(Critical)

Critical 455.24 (Asymptomatic)(Mild)(Moderate)(Severe)

Aspartate aminotransferase Asymptomatic 349.18 (Mild)(Moderate)(Severe)(Critical)

Mild 449.1 (Asymptomatic)(Moderate)(Severe)(Critical)

Moderate 610.81 (Asymptomatic)(Mild)(Severe)(Critical)

Severe 780.94 (Asymptomatic)(Mild)(Moderate)(Critical)

Critical 875.14 (Asymptomatic)(Mild)(Moderate)(Severe)

Alanine aminotransferase Asymptomatic 375.15 (Mild)(Moderate)(Severe)(Critical)

Mild 580.68 (Asymptomatic)(Moderate)(Severe)(Critical)

Moderate 654.38 (Asymptomatic)(Mild)(Severe)(Critical)

Severe 718.19 (Asymptomatic)(Mild)(Moderate)

Critical 764.47 (Asymptomatic)(Mild)(Moderate)

Albumin Asymptomatic 681.11 (Mild)(Moderate)(Severe)(Critical)

Mild 855.7 (Asymptomatic)(Moderate)(Severe)(Critical)

Moderate 589.07 (Asymptomatic)(Mild)(Severe)(Critical)

Severe 446.21 (Asymptomatic)(Mild)(Moderate)(Critical)

Critical 378.11 (Asymptomatic)(Mild)(Moderate)(Severe)

Total bilirubin Asymptomatic 426.31 (Severe)(Critical)

Mild 380.68 (Moderate)(Severe)(Critical)

Moderate 466.07 (Mild)(Critical)

Severe 514.78 (Asymptomatic)(Mild)(Critical)

Critical 598.85 (Asymptomatic)(Mild)(Moderate)(Severe)

Direct bilirubin Asymptomatic 259.64 (Moderate)(Severe)(Critical)

Mild 300.67 (Moderate)(Severe)(Critical)

Moderate 437.07 (Asymptomatic)(Mild)(Severe)(Critical)

Severe 572.01 (Asymptomatic)(Mild)(Moderate)(Critical)

Critical 681.14 (Asymptomatic)(Mild)(Moderate)(Severe)

***Inflammatory Markers***

Lactate dehydrogenase Asymptomatic 381.74 (Moderate)(Severe)(Critical)

Mild 318.96 (Moderate)(Severe)(Critical)

Moderate 564.2 (Asymptomatic)(Mild)(Severe)(Critical)

Severe 816.59 (Asymptomatic)(Mild)(Moderate)(Critical)

Critical 994.31 (Asymptomatic)(Mild)(Moderate)(Severe)

Serum ferritin Asymptomatic 270.15 (Mild)(Moderate)(Severe)(Critical)

Mild 350.44 (Asymptomatic)(Moderate)(Severe)(Critical)

Moderate 601.55 (Asymptomatic)(Mild)(Severe)(Critical)

Severe 840.6 (Asymptomatic)(Mild)(Moderate)(Critical)

Critical 932.24 (Asymptomatic)(Mild)(Moderate)(Severe)

Serum procalcitonin Asymptomatic 258.73 (Mild)(Severe)(Critical)

Mild 178.59 (Asymptomatic)(Moderate)(Severe)(Critical)

Moderate 300.73 (Mild)(Severe)(Critical)

Severe 437.5 (Asymptomatic)(Mild)(Moderate)(Critical)

Critical 496.22 (Asymptomatic)(Mild)(Moderate)(Severe)

D-dimer Asymptomatic 288.64 (Mild)(Severe)(Critical)

Mild 202.72 (Asymptomatic)(Moderate)(Severe)(Critical)

Moderate \ 339.53 (Mild)(Severe)(Critical)

Severe 487.5 (Asymptomatic)(Mild)(Moderate)(Critical)

Critical 582.7 (Asymptomatic)(Mild)(Moderate)(Severe)
